# Multiclass classification of autofluorescence images of oral cavity lesions based on quantitative analysis

**DOI:** 10.1371/journal.pone.0228132

**Published:** 2020-02-04

**Authors:** Ming-Jer Jeng, Mukta Sharma, Ting-Yu Chao, Ying-Chang Li, Shiang-Fu Huang, Liann-Be Chang, Lee Chow

**Affiliations:** 1 Department of Electronic Engineering, Chang Gung University, Taoyuan, Taiwan; 2 Department of Otolaryngology-Head and Neck Surgery, Chang Gung Memorial Hospital, Linkou, Taiwan; 3 Green Technology Research Center, Chang Gung University, Taoyuan, Taiwan; 4 Department of Public Health, Chang Gung University, Taoyuan, Taiwan; 5 Department of Physics, University of Central Florida, Orlando, FL, United States of America; University of California, Davis, UNITED STATES

## Abstract

**Background:**

Oral cancer is one of the most common diseases globally. Conventional oral examination and histopathological examination are the two main clinical methods for diagnosing oral cancer early. VELscope is an oral cancer-screening device that exploited autofluorescence. It yields inconsistent results when used to differentiate between normal, premalignant and malignant lesions. We develop a new method to increase the accuracy of differentiation.

**Materials and methods:**

Five samples (images) of each of 21 normal mucosae, as well as 31 premalignant and 16 malignant lesions of the tongue and buccal mucosa were collected under both white light and autofluorescence (VELscope, 400-460 nm wavelength). The images were developed using an iPod (Apple, Atlanta Georgia, USA).

**Results:**

The normalized intensity and standard deviation of intensity were calculated to classify image pixels from the region of interest (ROI). Linear discriminant analysis (LDA) and quadratic discriminant analysis (QDA) classifiers were used. The performance of both of the classifiers was evaluated with respect to accuracy, precision, and recall. These parameters were used for multiclass classification. The accuracy rate of LDA with un-normalized data was increased by 2% and 14% and that of QDA was increased by 16% and 25% for the tongue and buccal mucosa, respectively.

**Conclusion:**

The QDA algorithm outperforms the LDA classifier in the analysis of autofluorescence images with respect to all of the standard evaluation parameters.

## Introduction

Oral cancer is the sixth common malignancy globally; it is closely associated with smoking, drinking alcohol, chewing tobacco and chewing betel quid. The most common histology of oral cancer is squamous cell carcinoma [1]. In males, it is the most commonly found cancer in the head and neck, accounting for 90% of the 300,000 newly diagnosed oral malignancies every year [2]. According to Stewart [3], about 60% of the new oral cancer cases and 68% of the deaths associated with oral cancer are reported in Asia. In Taiwan, oral cancer is the fifth most common cause of death and the fourth most common among males [4]. Oral potentially malignant disorders (OPMDs) are mucosal lesions that have high potential to develop oral cavity squamous cell carcinomas (OCSCCs), including leukoplakia, erythroplakia, erythroleukoplakia, and submucosal fibrosis [5]. OCSCCs are typically diagnosed late, resulting in an overall five-year survival rate of 50% [6]. The early detection and timely treatment of premalignancy may prevent the transformation of OPMDs into oral cancer [7]. During this transformation process, tissue structures in the squamous epithelium and metabolism are distorted [8]. Collagen and elastin are degraded during the tumor development and invasion. This distortion is observed in histopathological examinations [9, 10]. The most frequently used method for early diagnosis is oral screening [11–13]. Although biopsies and histopathological examinations are the gold standard for diagnosing oral cancer [14], biopsy is an invasive and time consuming. It requires an incision in the tissues. These shortcomings have led clinicians to shift to non-invasive techniques such as vital staining, light-based detection and the use of optical diagnostic techniques [15].

This work focuses on a non-invasive and light-based detection device, VELscope, which exploits autofluorescence. Autofluorescence is the natural emission of light by biological structures. These structures assimilate light and can be differentiated by the light that originates from artificially added fluorescent markers. These markers become fluorescent upon excitation by light with wavelength 375-460 nm [16, 17]. Autofluorescence phenomena can easily be used to detect metabolic transformation in tissue structures and are therefore useful in screening for oral cancer [11]. In this study, premalignant and malignant lesions are differentiated to avoid the need for invasive biopsies, this non-invasive technique is better used for surveillance to determine whether a biopsy is needed but these methods can never replace the biopsy.

The VELscope (Visually Enhanced Lesion Scope) is a handheld device that increases the visibility of the oral membrane abnormalities by activating tissue fluorescence. It uses direct tissue autofluorescence with a wavelength of blue light (between 400 nm and 460 nm) to enhance the visibility of oral mucosal abnormalities, which are never visible under white light [18]. At these wavelengths, the normal oral mucosa is associated with a pale green fluorescence as viewed through a filter and abnormal tissue is associated with a loss of autofluorescence and appears dark. Many works [19–26] have evaluated the efficacy of the VELscope by direct comparing VELscope results with the biopsy reports. VELscope has been shown to have high sensitivity and to assist in the detection of oral lesions. However, it can not effectively differentiate between high-risk and low-risk lesions. Awan [19] demonstrated the relatively high sensitivity, 84% and a low specificity 15%, in differentiating high-risk lesions from benign lesions. Similarly, Ganga [26] obtained sensitivity and specificity values of 76% and 66.29%, respectively. They suggested that VELscope has reasonable sensitivity, but yields many false-positive results. Huang et al. [27] proposed the use of quantitative analysis(quadratic discriminant analysis, QDA) to quantify the classification of VELscope images (autofluorescence images) by their intensity and heterogeneity. They used QDA as a method of discriminant analysis classification to differentiate between normal and abnormal (malignant/premalignant) lesions of oral mucosa. They successfully differentiated between abnormal and normal lesions with higher specificity and good sensitivity. One major limitation of their study was evident in differentiating between malignant and premalignant lesions. Therefore, in this work, three groups of patients were recruited, having normal, premalignant and malignant lesions. Information in the image was normalized first and then linear discriminant analysis (LDA) and QDA were used for classification.

## Materials and methods

### Patients and samples

This study was approved by the Institutional Review Board (IRB) of Chang Gung Medical Foundation (IRB No: 201800420B0), Taiwan. It was performed in the Department of Otolaryngology-Head and Neck Surgery, with the written and informed consent of the enrolled participants. Clinical data and pathological reports were collected at Chang Gung Hospital for analysis. The patients with malignancy and premalignancy received a biopsy after the VELscope autofluorescence images were captured. Thirty-one and sixteen patients had premalignant and malignant lesions, respectively. Images of the tongues 11 healthy students at Chang Gung University and of the buccal mucosa of 10 such students were obtained. None of these healthy people habitually smoked, consumed alcohol or chewed betel quid. A total of 340 (68*5) images were analyzed, as shown in Table 1. Table 2 summarizes the demographics of the 47 patients who were classified by their lesions at subsites of the tongue and buccal mucosa. The tongues had 11 premalignant and eight malignant lesions and the buccal mucosae had 20 premalignant and eight malignant lesions.

**Table 1:** Total fluorescence images studied under VELscope.

	Total images	Tongue	Buccal mucosa
Normal	105	55 (11*5)	50 (10*5)
Premalignant	155	55 (11*5)	100 (20*5)
Malignant	80	40 (8*5)	40 (8*5)

**Table 2:** Patient demographics.

Characteristic	Premalignant	Malignant
Age (mean±SD)	52.8 ± 12.1	62.8 ± 11.2
Gender(M: F)	26: 5	13: 3
**location**		
Tongue	11 (35.5%)	8 (50%)
Buccal mucosa	20 (64.5%)	8 (50%)
**Tumor Stage**		
T1		5 (31.3%)
T2		7 (43.8%)
T3		0 (0%)
T4		4 (25%)
**Pathology**		
Parakeratosis	6 (19.4%)	
Mild Dysplasia	19 (61.3%)	
Moderate Dysplasia	1 (3.2%)	
Verrucous Hyperplasia	5 (16.1%)	

The premalignant lesions included leukoplakia and erythroplakia, which have higher risks of malignant transformation. The malignant lesions were all squamous cell carcinoma, as established by the biopsies. Five autofluorescence images were obtained under white and blue light at various angles and intensities in each case to increase the accuracy of our analysis. The images under blue light were captured using a *VELscope_*VX*_* (LED Dental and Apteryx, Atlanta Georgia, USA) with an iPod (Apple, Atlanta Georgia, USA) touch, which had eight million pixels.

### Methodology

**Fig 1:**
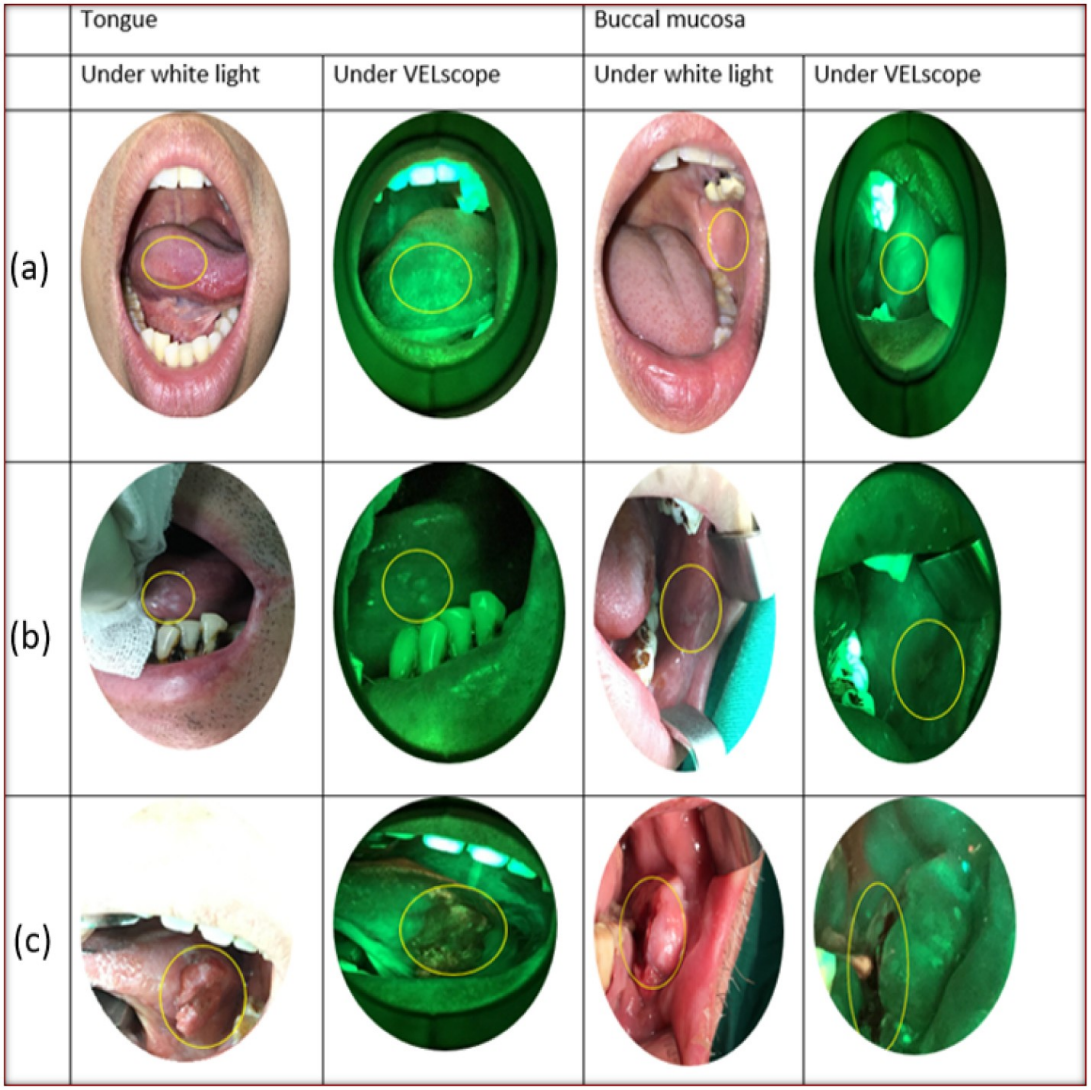
The white light and VELscope autofluorescence images of the tongue and buccal mucosa in selected ROI with (a) Normal, (b) Premalignant and (c) Malignant lesion.

**Fig 2:**
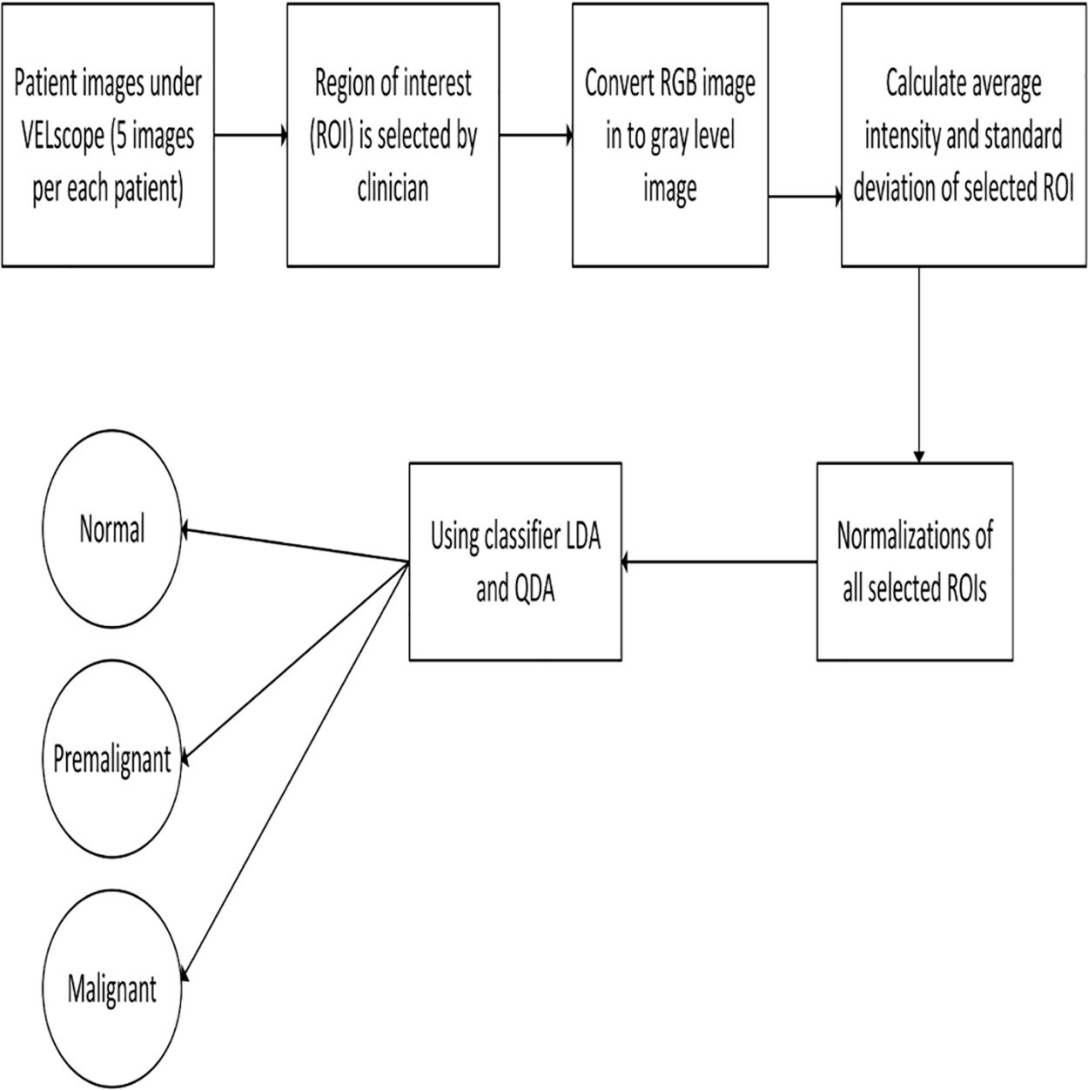
Block diagram of proposed methodology.

The clinician(Dr. Huang, S.F.) identified the region of interest(ROI) was selected within the lesion(premalignant/malignant) in each image. The analysis considered the same ROIs in the images obtained from the healthy persons. As shown in Fig 1, in the images of the tongue and buccal mucosa, the ROI is shown in a circle. The RGB images are converted into the gray-level images. The reduction of complexity (from 3D pixel to 1D pixel) of the calculation without changing the intensity of the image is important consideration. The following steps were taken and shown in Fig 2.

- Convert the RGB image into a gray-level image.
- Calculate the average intensity and standard deviation of the selected ROI.
- Normalization of ROI by calculating the normalized average intensity and standard deviation of intensity.
- Feed the above parameters into the LDA and QDA classifier to identify normal, premalignant and malignant lesions.

Under VELscope, the intensity of normal lesions is commonly higher than that of malignant lesions. In the work of Ganga [26], three fluorescence phenomena were observed under VELscope; they were FVI(fluorescence visualization increase), FVR(fluorescence visualization retained) and FVL(fluorescence visualization loss). All three phenomena were observed in the obtained images. The normal mucosa appears as pale green and fluorescence visualization is retained, so the mucosa is referred to as FVR. The malignant lesions exhibit FVL due to a loss of autofluorescence. The premalignant lesions exhibit either FVI or FVL. The images under VELscope were associated with green color. Fluorescence is the emission of light, causing energy to be lost. The tissue absorbs blue light but emits green light. Therefore, a quantitative analysis of green pixel values was performed, as described below. The average intensity and standard deviation of intensity provide information on the brightness and heterogeneity of selected ROIs. The surface of a malignant lesion was not as homogeneous as that of normal mucosa. Therefore, the standard deviation was calculated along with the intensity to determine the heterogeneity of each ROI. Eqs 1 and 2 give the mean intensity (*μ*) of an image and the standard deviation of intensity (*σ*). Where *A*_*i*_ is the array of R, G, B or gray pixel values of in the ROI and N is the number of pixels in the ROI. To normalize the average intensity and the standard deviation, Eqs 3 and 4 are used, where (*μ*_*N*_) and (*σ*_*N*_) are the normalized average intensity and standard deviation of intensity, respectively. The *μ*_*ROI*_ and *σ*_*ROI*_ are the average intensity and standard deviation in the ROI, respectively.

$$\begin{array}{c} \begin{array}{c} \mu =\left(1/N\right)\sum_{i=1}^{N}A_{i} \end{array} \end{array}$$ (1)

$$\begin{array}{c} \begin{array}{c} \sigma =\sqrt{1/\left(N-1\right)\sum_{i=1}^{N}{\left|A_{i}-\mu \right|}^{2}} \end{array} \end{array}$$ (2)

$$\begin{array}{c} \begin{array}{c} \mu_{N}=\frac{\mu_{ROI}}{\mu } \end{array} \end{array}$$ (3)

$$\begin{array}{c} \begin{array}{c} \sigma_{N}=\frac{\sigma_{ROI}}{\sigma } \end{array} \end{array}$$ (4)

### Normalization

The normalized intensity and the standard deviation of intensity were used to neutralize the autofluorescence in the ROI, which have arisen from the parts of the image outside the ROI. Non-ROI and unwanted autofluorescence, which may affect the ROI in the image; it can include teeth, supporting device, prosthesis, or filling materials. Normalization eliminated the redundant data and increased the variability and separability of the classifiers. The results of the quantitative analysis are validated by comparing normalized and un-normalized data.

### Data analysis

Many statistical methods, such as principal component analysis (PCA), LDA and QDA are used in pattern discrimination or classification, dimension reduction and pattern recognition. PCA finds the directions of maximal variance and can be used only in two-class classification. LDA targets class separability and maximizes the component axis for class separation [28]. LDA is the enhanced version of PCA and is frequently used in medical imaging, especially in pathological examinations to find a linear combination of features that separates several categories of objects [29]. QDA is an extension of, and more flexible than, LDA. LDA assumes a single variance-covariance matrix over all classes whereas the QDA assumes different variance-covariance matrices for each class. LDA yields a linear boundary whereas QDA produces a quadratic boundary of the classifier, resulting in substantially greater variance because it is quadratic [30]. Both classifiers are very helpful in medical applications [31, 32]. Therefore, the effectiveness of both in distinguishing among normal, premalignant and malignant lesions is studied herein. QDA and LDA assume that every category of information exhibits multivariate Gaussian distributions. The equation for multivariate Gaussian distribution for class *n* is Eq 5, where *k* is a dimension; ∑_*n*_ is the covariance of class n and *μ*_*n*_ is the mean of class n. Eqs 6 and 7 are the LDA and QDA functions.

$$\begin{array}{c} \begin{array}{c} f_{n}\left(x\right)=1/\sqrt{{\left(2\pi \right)}^{k/2}{\left|\sum_{n}\right|}^{1/2}})e^{-1/2{\left(x-\mu_{n}\right)}^{T}\sum_{n}^{-1}\left(x-\mu_{n}\right)} \end{array} \end{array}$$ (5)

$$\begin{array}{c} \begin{array}{c} \delta_{n}\left(x\right)=-\frac{1}{2}log\left|\sum_{n}\right|-\frac{1}{2}{\left(x-\mu_{n}\right)}^{T}\sum_{n}^{-1}\left(x-\mu_{n}\right)+log\pi_{n} \end{array} \end{array}$$ (6)

$$\begin{array}{c} \begin{array}{c} \delta_{n}\left(x\right)=x^{T}\sum^{-1}\mu_{n}-\frac{1}{2}\mu_{n}^{T}\sum^{-1}\mu_{n}+log\pi_{n} \end{array} \end{array}$$ (7)

Sensitivity and specificity are commonly used parameters in binary classification. However, multiclass classification is used herein. The performance of each classifier for tongue and buccal mucosa was evaluated using such parameters as precision, recall, f1-score and accuracy. Precision is the positive predictive value (PPV), which is defined as the number of relevant cases as a proportion of the retrieved cases. Recall is the true positive rate (TPR) or sensitivity, which is defined as the number of relevant cases that have been retrieved divided by the total number of relevant cases. The F1-score is the harmonic mean of precision and recall, accuracy is the proportional precision in a classification system. The definitions are given by Eqs 8 to 11. Here, TP is True Positive (an abnormal lesion is categorized correctly as abnormal); TN is True Negative (a normal is categorized correctly as normal); and FP is False Positive (a normal is categorized wrongly as abnormal); FN is False Negative (an abnormal is categorized wrongly as normal). The confusion table provides the numbers of correct and incorrect predictions. Abnormalities include any premalignant or malignant lesion. Therefore, TP and TN correspond to true or correct classification and FP and FN correspond to false or incorrect classification.

$$\begin{array}{c} \begin{array}{c} Accuracy=\frac{TP+TN}{TP+TP+FN+FP} \end{array} \end{array}$$ (8)

$$\begin{array}{c} \begin{array}{c} Precision=\frac{TP}{TP+FP} \end{array} \end{array}$$ (9)

$$\begin{array}{c} \begin{array}{c} Recall=\frac{TP}{TP+FN} \end{array} \end{array}$$ (10)

$$\begin{array}{c} \begin{array}{c} F1-score=\frac{2*Precision*Recall}{Precision+Recall} \end{array} \end{array}$$ (11)

## Results

All the participants were registered between July 2017 and September 2018, having provided written and informed consent. Biopsies confirmed that 16 and 31 patients had malignant and premalignant, respectively. Twenty-one healthy participants were enrolled. Table 1 provides information about the patients. Fig 1 shows the white-light and autofluorescence images. The clinician (Dr. Huang S.F.) selected all ROIs. The intensity and standard deviation of intensity of all autofluorescence images were recorded and analyzed. The standard deviations revealed the heterogeneity of lesions. The intensity and standard deviation of intensity among adjacent non-ROIs were recorded and used in normalization. Intensity and standard deviation of intensity were analyzed using multiclass classification algorithms.

For multiclass classification, LDA and QDA classifiers were used. To prevent over-fitting, 75% of the data were used in training and the remaining 25% were used in testing. k-Fold cross-validation (k = 2) were used with the LDA and QDA classifiers to categorize the lesions in autofluorescence images. In k-fold cross-validation, the data were divided into k equal folds. Then, the algorithm was trained for using k-1 folds, while the remaining fold was used as the test set. The performance was aggregated across k folds. Each classifier yielded confusion and performance tables. Decision boundary curves were developed separately for the tongue and buccal mucosa. Each row of the confusion table represented instances in a predicted class, and each column represented instances in an actual class. The diagonal and non-diagonal elements represented true/correct and false/incorrect predictions, respectively. Normalized and un-normalized data were analyzed. The un-normalized data yielded poorer differentiation than the normalized data, indicating that the autofluorescence of the ROI was influenced by the non-ROI regions. Precision and recall are the two important parameters in multiclass classification. Accuracy is not an adequate parameter for evaluating the performance evaluation of a predictive model, owing to the accuracy paradox [33]. Therefore, precision and recall are used as the critical performance parameters in our analysis. Both LDA and QDA had the potential to differentiate the three classes (N, M, and PM) but the QDA classifier yielded better results than the LDA perhaps because the latter is data-independent, whereas the former is data-dependent.

**Fig 3:**
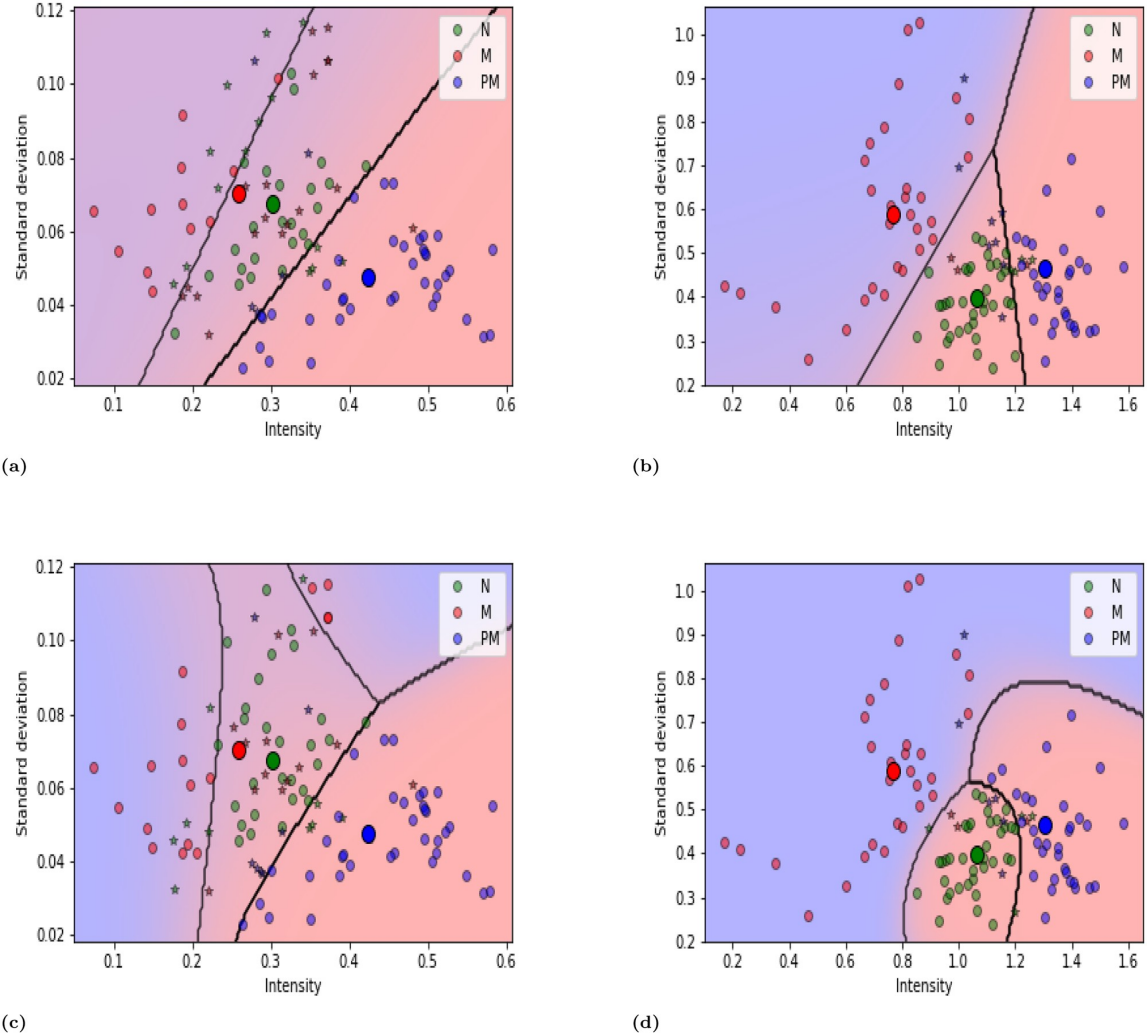
For tongue (a) Un-normalized and (b) Normalized data were plotted using LDA classifier, whereas (c) Un-normalized and (d) normalized data were plotted using QDA classifier. The misclassification exhibited as a star and the big dark circles represented the mean of each class. The results showed that the linear boundary ((a) and (b)) and quadratic boundary ((c) and (d)) can discriminate among normal (N), malignant (M) and premalignant lesions (PM).

One hundred and fifty images of the tongue subsite were obtained, of which 112 were used for training and 38 were used for testing. LDA yielded a total of 74 (24+12+38) and 97 (36+31+30) true or correctly classified cases, and 38 (15+18+5) and 15 (4+3+8) false or incorrectly classified cases, with un-normalized and normalized training data, respectively, as shown in Table 3. QDA yielded a total of as 81 (28+17+36) and 99 (36+31+32) true or correctly classified cases, and 31 (11+13+7) and 13 (4+3+6) false or incorrectly classified cases, with un-normalized and normalized training data, respectively. Similar results were obtained with testing data [Table 3]. In evaluating the LDA, normalized training and testing data yielded 21% and 27% greater precision than un-normalized data, respectively [Table 4]. For QDA, the corresponding values were 16% and 20%, respectively. QDA with normalized testing and training data differentiated between tongue lesions with 86% and 88% accuracy, respectively; with un-normalized training and testing data, the accuracy rates were 72% and 68%, respectively. The decision boundary curves in Fig 3(a) to 3(d) revealed more misclassification with un-normalized data than with normalized data. Fig 3(a) and 3(b) show linear boundaries among three classes, based on differences in intensity and standard deviation of intensity, obtained using normalized and un-normalized data. Fig 3(c) and 3(d) show quadratic boundaries, which clearly differentiated the three categorizes.

**Table 3:** Confusion table of LDA and QDA for tongue.

**LDA**												
	**Un-normalized**						**Normalized**					
	**Training**			**Testing**			**Training**			**Testing**		
	N	M	PM	N	M	PM	N	M	PM	N	M	PM
N	24	10	5	8	5	3	36	0	4	12	0	3
M	17	12	1	7	3	0	2	31	1	0	6	0
PM	4	1	38	1	1	10	6	2	30	3	1	13
**QDA**												
	**Un-normalized**						**Normalized**					
	**Training**			**Testing**			**Training**			**Testing**		
	N	M	PM	N	M	PM	N	M	PM	N	M	PM
N	28	6	5	13	0	3	36	1	3	12	1	2
M	12	17	1	7	3	0	2	31	1	0	6	0
PM	7	0	36	2	0	10	4	2	32	1	1	15

**Table 4:** Performance table of LDA and QDA for tongue.

**LDA**												
	**Un-normalized**						**Normalized**					
	**Training**			**Testing**			**Training**			**Testing**		
	**Precision**	**Recall**	**f1-score**	**Precision**	**Recall**	**f1-score**	**Precision**	**Recall**	**f1-score**	**Precision**	**Recall**	**f1-score**
N	0.53	0.62	0.57	0.50	0.50	0.50	0.82	0.90	0.86	0.80	0.80	0.80
M	0.52	0.40	0.45	0.33	0.30	0.32	0.94	0.91	0.93	0.86	1.00	0.92
PM	0.86	0.86	0.87	0.77	0.83	0.80	0.86	0.79	0.82	0.81	0.76	0.79
$\frac{avg}{total}$	0.65	0.66	0.65	0.54	0.55	0.54	0.86	0.86	0.86	0.81	0.81	0.81
**QDA**												
	**Un-normalized**						**Normalized**					
	**Training**			**Testing**			**Training**			**Testing**		
	**Precision**	**Recall**	**f1-score**	**Precision**	**Recall**	**f1-score**	**Precision**	**Recall**	**f1-score**	**Precision**	**Recall**	**f1-score**
N	0.60	0.72	0.65	0.59	0.81	0.68	0.86	0.90	0.88	0.92	0.80	0.86
M	0.74	0.57	0.64	1.00	0.30	0.46	0.91	0.91	0.91	0.75	1.00	0.86
PM	0.86	0.84	0.85	0.77	0.83	0.80	0.89	0.84	0.86	0.88	0.88	0.88
$\frac{avg}{total}$	0.72	0.72	0.73	0.66	0.68	0.75	0.88	0.88	0.88	0.86	0.86	0.87

**Fig 4:**
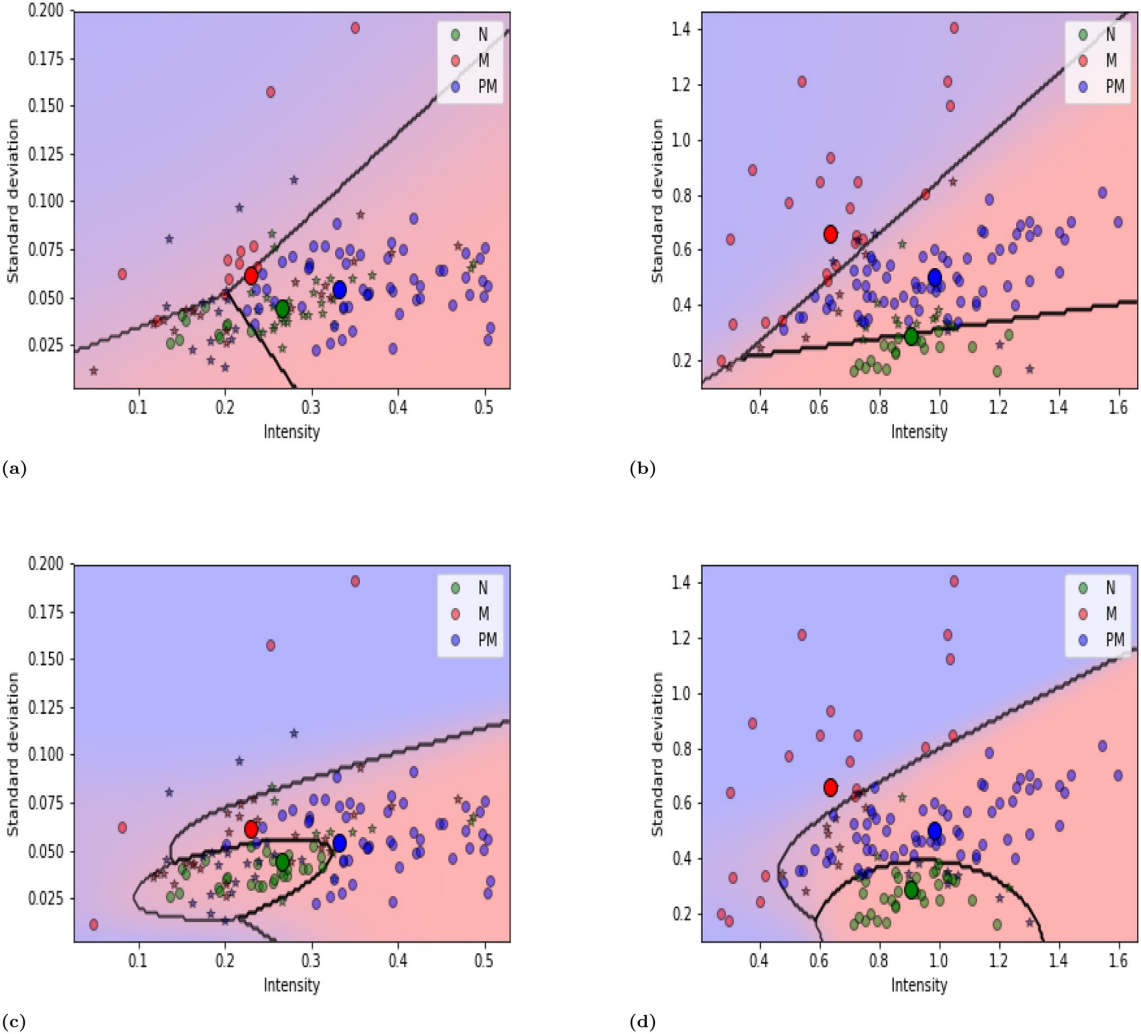
For buccal mucosa (a) Un-normalized and (b) Normalized data were plotted using LDA classifier, whereas (c) Un-normalized and (d) normalized data were plotted using QDA classifier. The misclassification exhibited as a star and the big dark circles represented the mean of each class. The results showed that the linear boundary ((a) and (b)) and quadratic boundary ((c) and (d)) can discriminate among normal (N), malignant (M) and premalignant lesions (PM).

For the buccal mucosa, 142 and 48 images (total 190 images) were used for training and testing data, respectively. LDA yielded a total of 78 (10+11+57) and 111 (21+22+68) true or correctly classified cases and 64 (30+ 19+ 15) and 31 (17+7+7) false or incorrectly classified cases, using un-normalized and normalized training data, respectively, as shown in Table 5. QDA yielded 85 (29+ 4+ 52) and 120(35+20+65) true or correctly classified cases and 57 (11+26+20) and 22 (3+9+10) false or incorrectly classified cases, using un-normalized and normalized training data, respectively. Similar results were obtained using the testing data [Table 5]. For LDA, the normalized training and testing data yielded 25% and 9% greater precision, respectively, than the un-normalized data [Table 6]. For QDA, the corresponding values are 28% and 16%, respectively, as shown in Table 6. The QDA differentiated tongue lesions with 72% and 84% accuracy with normalized testing and training data, respectively, and 56% and 59% accuracy with un-normalized training and testing data, respectively. In Fig 4(a) to 4(d), the decision boundary curves reveal more misclassification with un-normalized data than with normalized data. Fig 4(a) and 4(b) show linear boundaries among three classes, based on differences in intensity and standard deviation of intensity, with un-normalized and normalized data. Fig 4(c) and 4(d) show quadratic boundaries, which clearly differentiated among the three categorizes.

**Table 5:** Confusion table of LDA and QDA for buccal mucosa.

**LDA**												
	**Un-normalized**						**Normalized**					
	**Training**			**Testing**			**Training**			**Testing**		
	N	M	PM	N	M	PM	N	M	PM	N	M	PM
N	10	2	28	3	0	7	21	0	17	4	0	8
M	10	11	9	4	1	5	1	22	6	1	6	4
PM	8	7	57	5	1	22	3	4	68	3	3	19
**QDA**												
	**Un-normalized**						**Normalized**					
	**Training**			**Testing**			**Training**			**Testing**		
	N	M	PM	N	M	PM	N	M	PM	N	M	PM
N	29	1	10	7	0	3	35	0	3	9	0	3
M	13	4	13	2	4	4	0	20	9	0	8	3
PM	15	5	52	11	1	16	8	2	65	5	2	18

**Table 6:** Performance table of buccal mucosa.

**LDA**												
	**Un-normalized**						**Normalized**					
	**Training**			**Testing**			**Training**			**Testing**		
	**Precision**	**Recall**	**f1-score**	**Precision**	**Recall**	**f1-score**	**Precision**	**Recall**	**f1-score**	**Precision**	**Recall**	**f1-score**
N	0.36	0.25	0.29	0.25	0.30	0.27	0.84	0.55	0.67	0.50	0.33	0.40
M	0.55	0.37	0.44	0.50	0.10	0.17	0.85	0.76	0.80	0.67	0.55	0.60
PM	0.61	0.79	0.69	0.65	0.79	0.71	0.75	0.91	0.82	0.61	0.76	0.68
$\frac{avg}{total}$	0.52	0.54	0.52	0.5	0.54	0.53	0.77	0.78	0.79	0.59	0.60	0.59
**QDA**												
	**Un-normalized**						**Normalized**					
	**Training**			**Testing**			**Training**			**Testing**		
	**Precision**	**Recall**	**f1-score**	**Precision**	**Recall**	**f1-score**	**Precision**	**Recall**	**f1-score**	**Precision**	**Recall**	**f1-score**
N	0.51	0.72	0.60	0.35	0.70	0.47	0.81	0.92	0.86	0.64	0.75	0.69
M	0.40	0.13	0.20	0.80	0.40	0.53	0.91	0.69	0.78	0.80	0.73	0.76
PM	0.69	0.72	0.71	0.70	0.57	0.63	0.84	0.87	0.86	0.75	0.72	0.73
$\frac{avg}{total}$	0.56	0.59	0.57	0.57	0.56	0.64	0.84	0.84	0.84	0.73	0.72	0.73

**Fig 5:**
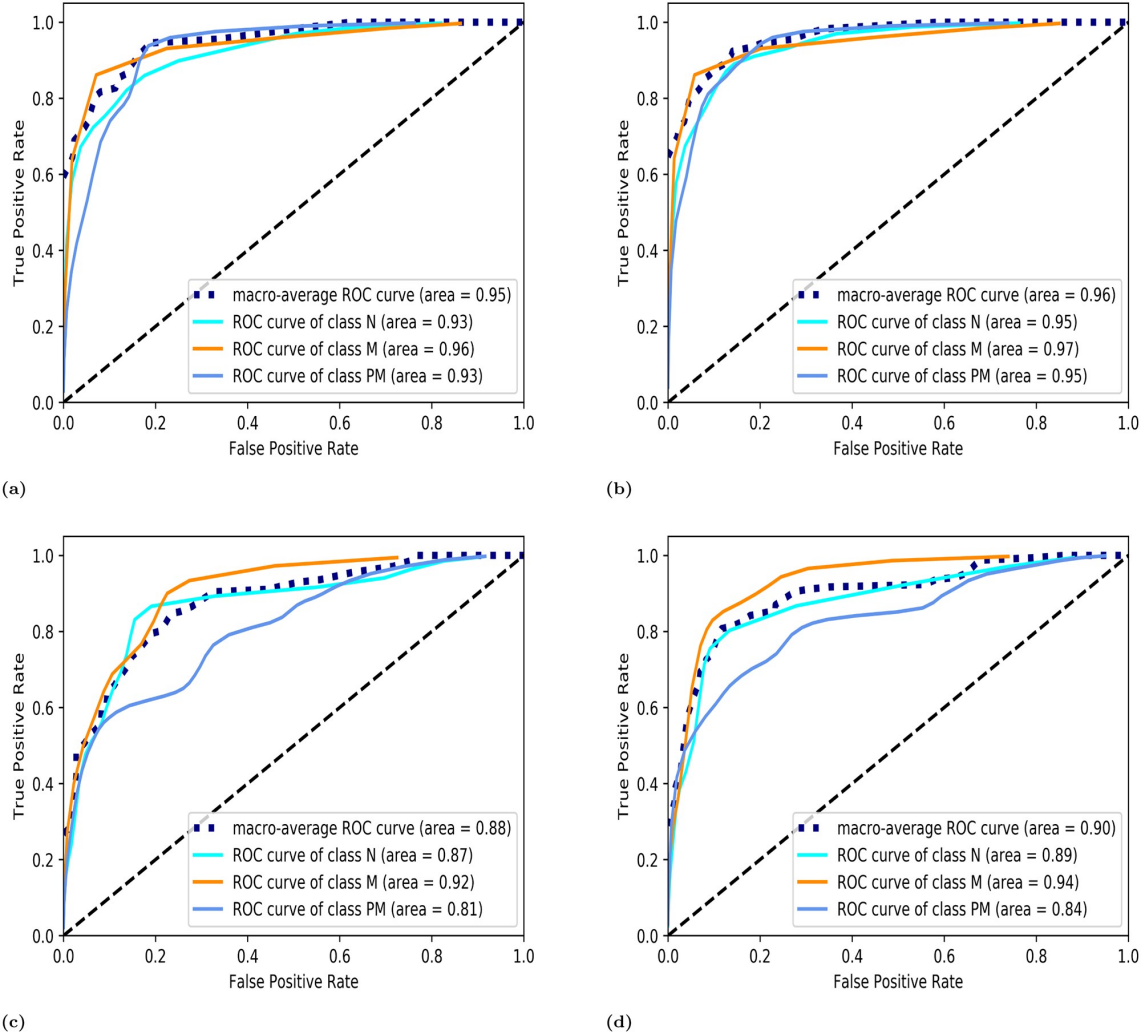
ROC curves were plotted (a) For normalized data of tongue using LDA and (b) QDA classifier (c) For normalized data of buccal mucosa using LDA and (d) QDA classifier. Where N is normal, PM is premalignant and M is malignant. Macro-average ROC curve gives the average of the three classes.

To visualize the multiclass classification performance, ROC (Receiver Operating Characteristic) curves were generated for both classifiers and drawn between the true positive rate and the false positive rate (1-specificity). An ROC curve is a probability curve and the AUC (Area Under the Curve) represents the degree or measure of separability. This can distinguish among classes. In Fig 5, ROC curves are obtained using the LDA and QDA classifiers and normalized data for the tongue and buccal mucosa. They show that the classification separability achieved using QDA exceeds that achieved using LDA. This study focused on the early diagnosis of oral cancer to identify premalignancy. For the tongue, a premalignant classification separability of 93% was achieved using the LDA classifier and of 95% using the QDA classifier, as shown in Fig 5(a) and 5(b), respectively. For the buccal mucosa, a premalignant classification separability of 81% was achieved using the LDA classifier and of 84% using the QDA classifier, as shown in Fig 5(c) and 5(d), respectively.

## Discussion

Autofluorescence is used in a fast and non-invasive method for detecting malignant and premalignant lesions. Many new diagnostic techniques and instruments are routinely used; they include Vizi-Lite (Zila Pharmaceuticals, Phoenix, AZ), Identafi (DentalEZ, PA, USA), Narrow band imaging (NBI; Olympus Medical Systems Corporation, Tokyo, Japan) and VELscope (LED Medical Diagnostics Inc., Burnaby Canada) [34, 35]. Vizi-Lite is an old device that is based on chemical autofluorescence. Identafi uses multispectral fluorescence to facilitate intraoral examination. NBI is an endoscopic technique to enhance the visualization of oral mucosal abnormalities and underlying vasculature. It uses a specific wavelength of light to detect the vasculature in submucosa [36, 37]. Scientists from the British Columbia Cancer Agency (BCCA) developed the VELscope, in which an extrinsic light source (400-460nm) is used to excite endogenous fluorophores. When this exogenous light interacts with the oral mucosa, the fluorophores absorb a photon of this light and emit a lower-energy photon; this process is known as fluorescence. Under this light, normal oral mucosa emits green autofluorescence, whereas the abnormal mucosa appears dark owing to the loss of autofluorescence(LAF) [38]. The two most diagnostically important endogenous fluorophores are Nicotinamide Adenine Dinucleotide (NADH) and Flavin Adenine Dinucleotide (FAD). They are used to monitor dramatic metabolic changes in cells and tissues. The oxidation-reduction (redox) ratio or NADH/FAD is used to measure cellular metabolism [39]. Each fluorophore has a specific excitation and emission spectrum. Fluorophores are not distributed uniformly in tissues; rather, each tissue contains a greatly varying concentration of fluorophores [40]. Although many optical diagnostic methods exist, none has yet been identified as having the best identification rate.

This work focused on diagnosing oral lesions in the preliminary stage before they become malignant. It presented a multiclass classification approach, which successfully differentiates among premalignant, malignant and normal tissues. VELscope autofluorescence images were captured and the normalized average intensities and standard deviation of intensities in ROIs were calculated. Intensity-based classification alone is not good enough. Huang TT et al. developed a method that VELscope autofluorescence images by QDA [27]. They calculated the intensity and heterogeneity of the ROI in the images. They used the QDA classifier to distinguish normal tissues from the premalignant and malignant lesions. They effectively classified normal and abnormal tissues. However, they did not differentiate between the premalignancies and the malignancies. In the present study, in contrast, premalignant and malignant lesions were successfully differentiated, as were normal and abnormal tissues, using a novel normalization technique. Currently, no quantitative analytical method of multiclass classification method exists for diagnosing oral cancer. Our method is helpful for detecting early-stage oral cancer. The detection of more OPMDs corresponds to a higher survival rate. The five-year survival rate for patients with late-stage oral cancer is only 20% and that for those with early-stage premalignant-stage oral cancer is about 82% [41].

In this work, most premalignant cases are related to leukoplakia and erythroplakia, which are the most common OPMDs. The fluorescent intensity of leukoplakia lesions exceeded that of the normal tissues, owing to FVI autofluorescence. Erythroleukoplakia lesions are associated with both FVL and FVI autofluorescences. They yielded a mixed autofluorescence signal and appeared dark brown in images. Erythroplakia is associated with a loss of fluorescence while leukoplakia in the periphery presents as FVI. Erythroleukoplakia can present as dark brown under VELscope. Malignant lesions usually exhibit a loss of autofluorescence (fluorescence visualization loss or FVL) and have appeared dark black. Sometimes, malignant lesions did not appear as darker they were formed by transformation from leukoplakia, but they then fluoresced like a premalignant lesion. Therefore, different types of lesion do not exhibit the same autofluorescence because they vary in location and morphology [42]. Quantitative analysis is required because VELscope alone does not provide appropriate useful information about the type of lesion. Heterogeneity is also important in analyzing an image, as it can be used to distinguish between normal and abnormal lesions. Therefore, variations in intensity and standard deviation of intensity were quantitatively analyzed for normal, premalignant and malignant lesions. The quantitative analysis involved multiclass classification with normalization. The two subsites (tongue and buccal mucosa) of oral mucosa yielded separate results, unlike in other research. The method successfully differentiated among normal (N), premalignant (PM) and malignant (M) lesions. LDA and QDA were used for classification. In this study, intensity and standard deviation of intensity in the malignant lesions differed between the subsites. Generally, tumor lesions exhibited variation in intensity and heterogeneity owing to the different locations of the tumors, but for the tongue (Fig 3(b) and 3(d)), more of the tumors were in stage T1 than in stage T2 or T4 (as indicated in the patient demographics table). Therefore, the intensity and standard deviation vary greatly on tumor subsites on the tongue. For the buccal mucosa (Fig 4(b) and 4(d)), more of the tumors were in stage T2 than in stage T1 or T4. Owing to the variation in the distribution of tumor stages, the standard deviation of intensity in malignant regions exceeded those in normal and premalignant regions. Image normalization has an integral role in any image based analysis and reduces variability among samples even when the experimental conditions are perfect [43]. The normalization technique that was used herein adequately improves the evaluation parameters and feature extraction without compromising the basic features in the images. The results of the quantitative analysis are validated by comparing normalized and un-normalized samples. We hope that this approach can further improve the differentiation between premalignant and malignant lesions. However, VELscope without quantitative analysis is not effective for such differentiation. The quantitative and multiclass classification approaches herein successfully distinguish premalignant lesions from normal and malignant lesions. Based on an earlier study, we can conclude that different parts of oral mucosa (tongue, buccal, gingiva, hard palate, soft palate) have different percentages of collagen and elastin [44]. Variations in collagen and elastin compositions among subsites of the oral mucosa are analyzed quantitatively. The quantitative analysis at a specific subsite cannot be generalized to all subsites of the oral mucosa. In this work, tongue and buccal mucosa were analyzed because they are the most frequent tumor subsites in patients in Taiwan owing to the popularity of smoking and the chewing of areca quid. All cases that involve the tongue involve the lateral subsite. The alteration at other subsites requires further investigation.

The first limitation of this study is that it is retrospective. A limited number of cases of premalignancy/malignancy were used. To increase sensitivity and specificity, a huge data set must be analyzed. Another inherent limitation of VELscope is that it cannot easily capture images of all interior subsites of the oral mucosa, including the hard palate and the retromolar.

This study provides a baseline definition of the boundary between surgical and tumor-free resection margin. This work also can help surgeons to determine the type of lesion. In the future, we will use other optical technologies to identify different stages (more than two) of oral cancer.

## Conclusion

Without quantitative analysis, VELscope cannot be used definitively to identify dysplastic tissue changes. This work applied normalization to autofluorescence images to calculate its normalized average intensity and standard deviation of its intensity. Multiclass classifiers (LDA and QDA) were used to generate decision boundary curves. QDA classifies normal, premalignant and malignant lesions with greater accuracy, precision, recall and f1-score, which have seldom been considered in the literature. Although these methods cannot completely replace biopsies, they are helpful to clinicians in detecting oral cancer early. In the future, we will introduce other classifiers with light sources with many wavelengths to increase the accuracy of detection.

## Supporting information

S1 Table Pathology report. (XLSX)

S2 Table Unnormalized buccal. (XLSX)

S3 Table Normalized buccal. (XLSX)

S4 Table Normalized tongue. (XLSX)

S5 Table Unnormalized tongue. (XLSX)
